# Stress, drink, leave: An examination of gender-specific risk factors for mental health problems and attrition among licensed attorneys

**DOI:** 10.1371/journal.pone.0250563

**Published:** 2021-05-12

**Authors:** Justin Anker, Patrick R. Krill

**Affiliations:** 1 Department of Psychiatry, University of Minnesota, Minneapolis, MN, United States of America; 2 Krill Strategies, LLC, Minneapolis, MN, United States of America; National Institue on Drug Abuse, UNITED STATES

## Abstract

Rates of mental illness and heavy alcohol use are exceedingly high in the legal profession, while attrition among women has also been a longstanding problem. Work overcommitment, work-family conflict, permissiveness toward alcohol in the workplace, and the likelihood of promotion are all implicated but have yet to be systematically investigated. Data were collected from 2,863 lawyers randomly sampled from the California Lawyers Association and D.C. Bar to address this knowledge gap. Findings indicated that the prevalence and severity of depression, anxiety, stress, and risky/hazardous drinking were significantly higher among women. Further, one-quarter of all women contemplated leaving the profession due to mental health concerns, compared to 17% of men. Logistic models were conducted to identify workplace factors predictive of stress, risky drinking, and contemplating leaving the profession. Overcommitment and permissiveness toward alcohol at work were associated with the highest likelihood of stress and risky drinking (relative to all other predictors) for both men and women. However, women and men differed with respect to predictors of leaving the profession due to stress or mental health. For women, work-family conflict was associated with the highest likelihood of leaving, while overcommitment was the number one predictor of leaving for men. Mental health and gender disparities are significant problems in the legal profession, clearly requiring considerable and sustained attention.

## Introduction

The United States legal profession is in the midst of a cultural reckoning related to the mental health and well-being of its members. Recent national reports indicate that lawyers suffer from exceedingly high rates of depression, anxiety, and substance misuse [1,2]. For example, in a large nationwide study of 12,825 licensed, currently practicing attorneys, 28% reported symptoms of depression, 23% indicated having mild to extremely severe stress, and 20.6% engaged in problematic drinking [1]. However, this problem extends beyond the individual lawyer and has the potential to impact not only clients but also the legal system more broadly. As a result, institutions and stakeholders have cast a critical eye on practices that contribute to poor mental health, including many of the attitudes and behaviors often considered synonymous with success in the legal profession, such as long hours and work overcommitment. There is a growing consensus that more needs to be done to improve the situation, and a movement has emerged to position mental health as a visible strategic priority for the legal profession. This has included the formation of national and state task forces (e.g., The National Task Force on Lawyer Well-Being), hundreds of large employers signing an ABA-sponsored pledge to reduce mental health and addiction problems (American Bar Association Well-Being Pledge), and a proliferation of media coverage [3,4].

As the extent of mental health problems is brought to light, it is also becoming apparent that these problems may not affect men and women equally. Reports have indicated that levels of anxiety and problematic drinking may be higher among women in the legal profession. Moreover, a very noticeable and serious gender disparity exists related to attorney attrition, with some reports estimating attrition rates for women to be 150% higher than men [5].

While the recent efforts to improve lawyer mental health have been a clear step in the right direction, what has not materialized is significant empirical evidence into the nature, scope, and causes of the mental health and substance use challenges lawyers face, as well as the gender disparities associated with each. The present study aims to address these vitally important objectives by identifying work-related factors predictive of three key challenges currently facing the legal profession: stress, substance misuse, and attrition. We focused on perceived stress as a primary psychopathology construct given the well-established role of stress as both a cause and consequence of depression and anxiety, which are exceedingly high among lawyers. Similarly, risky drinking was examined given the growing prevalence and severity of alcohol misuse within the legal profession. Finally, we focused on lawyer attrition, given that an exodus of highly skilled attorneys is occurring at an alarming rate, especially among women. We investigated the following work-related factors as predictors of these problems: overcommitment to work, an imbalance between effort and reward, work-family conflict, and workplace permissiveness toward alcohol.

By spotlighting these previously unexamined aspects of the attorney experience, our hope is to provide a foundation and catalyst for additional improvement of the legal profession.

## Methods

### Participants

#### Recruitment and random selection

The study design and protocol were reviewed by the University of Minnesota Institutional Review Board and deemed exempt from approval. An Exemption Determination was issued on March 20, 2020. Recruitment was coordinated in collaboration with the California Lawyers Association (“CLA”), a nonprofit, voluntary organization that includes the Sections of the State Bar of California and the California Young Lawyers Association, and the D.C. Bar, the largest unified bar in the United States and an organization which provides oversight structure to maintain ethical standards and Rules of Professional Conduct. An advertisement was included in newsletters sent by the D.C. Bar and CLA to their respective member lists and posted on their organization’s website. The advertisement provided a summary of the study, indicated that the survey was anonymous and that members would be randomly invited to participate in the study via email. Participants were randomly selected from a list of unique de-identified I.D.s supplied by the CLA and D.C. Bar. Each list contained approximately 98,000 IDs (196,000 total IDs). 40,000 IDs were randomly selected from each list (80,000 total) using the random sample function in the statistical platform R [6]. An email notification was sent to randomly selected D.C. Bar and CLA members on behalf of the researchers. Seven days following the email notification, study candidates received an email containing a link to a REDCap (Research Electronic Data Capture) survey. Clicking on the link directed participants to the survey’s informed consent page.

### Materials

#### Descriptive variables

*Demographics and work-related variables*. Information regarding age, race, relationship status, and whether respondents had children were collected. Additionally, information on the following work-related variables was collected from participants: the average number of hours worked per week, current position in the legal profession, and whether the current position involved litigation.

*Mental health variables*. Participants were asked if they had ever had a diagnosis of alcohol use disorder and whether they were a current, former, or lifetime abstainer of alcohol and drugs. Participants completed the Patient Health Questionaire-9 (PHQ-9) [7] and Generalized Anxiety Disorder-7 (GAD-7) [8] to assess the prevalence and severity of symptoms of depression and anxiety, respectively. For the PHQ-9, participant scores were grouped across the following 5 categories: None/Minimal (0–4), Mild (5–9), Moderate (10–14), Moderately Severe (15–19), and Severe (20–27). For the GAD-7, scores were grouped across the following 4 categories: None/Minimal (0–4), Mild (5–9), Moderate (10–14), and Severe (15–21). The total score on the Perceived Stress Scale (PSS) was used to assess how unpredictable, uncontrollable, and overloaded respondents found their lives. Scores on the PSS were grouped into Low (0–13), Moderate (14–26), and Severe (27–40) categories for analyses comparing. Scores on the Alcohol Use Disorders Identification Test (AUDIT-C) were used to assess risky drinking (women ≥ 3; men ≥ 4) and high-risk/hazardous drinking (women ≥ 4; men ≥ 5).

#### Predictor variables

Predictors of stress, substance misuse, and attrition were selected based on well-known aspects of the legal profession and were assumed to contribute to each outcome being examined. Those predictors included overcommitment to work, an imbalance between effort and reward, work-family conflict, and workplace permissiveness toward alcohol. We also examined the extent to which prospects of career growth in the form of promotion were associated with lower rates of stress, alcohol misuse, and thoughts of leaving the profession. Predictive modeling was conducted separately for women and men since gender disparities in the relationship between mental health and attrition have yet to be investigated despite a growing consensus of their existence.

*Effort-reward imbalance*, *overcommitment*, *and promotion*. The Effort-Reward Imbalance (ERI) Questionnaire [9] is comprised of 16 items and is used to determine if ERI and overcommitment are present in the workplace. The instrument consists of 16 items that measure effort, reward, and overcommitment on a four-point Likert scale (1 = Strongly Disagree, 2 = Disagree, 3 = Agree, 4 = Strongly Agree). The Effort-Reward Imbalance (ERI) ratio, Overcommitment, and Promotion subscales of the ERI Questionnaire were used to assess the imbalance between effort (meeting job demands) and reward, exhaustion, and being overwhelmed by work demands and the perceived prospects of promotion. With respect to the ERI ratio scale, a score above one reflects imbalance in the form of greater effort needed for reward, while a score below one reflects less effort needed for reward.

*Workplace permissiveness toward alcohol (Your Workplace)*. Five items from the Your Workplace questionnaire were used to assess the frequency of alcohol-related work activities in the participants’ workplace [10] e.g., “How many times in the last six months of your last position in the legal profession have some of your co-workers gone drinking off the job?” with the following response options: 1) never, 2) only once in the last six months, 3) 2–5 times in the last six months, 4) about once every 2 weeks, 5) about once a week, and 6) 2–4 times a week.

*Work-family conflict*. The degree to which work interfered with family life was assessed using three items from the Work-Family Conflict (WFC) subscale from the short version of the Copenhagen Psychosocial Questionnaire [11]. Participants rated items on a 4-point Likert-scale ranging from 1 (no, not at all) to 4 (yes, certainly).

*Accounting for COVID-19*. It is important to acknowledge that data collection for the study occurred during the COVID-19 pandemic. To control the pandemic’s collateral burden on the study outcomes, variables representing the degree to which stress and drinking changed since the beginning of the pandemic were entered into models as covariates. To this end, a single item assessing change due to COVID-19 was included at the end of the Perceived Stress Scale (PSS) (“Thinking back to before the COVID-19 pandemic, do you believe the frequency of these problems has remained the same, decreased, or increased?”) and the AUDIT (“Thinking back to before the COVID-19 pandemic, do you believe the frequency of your alcohol use has remained the same, decreased, or increased?”).

#### Outcome variables

*Stress*. We focused on total score on the 10-item PSS as a primary psychopathology construct, given its well-established correlation with psychiatric and physical disorders [12–14]. Consequently, participants who scored in the moderate to high range were grouped and compared to the low-stress group for logistic modeling.

*Risky drinking*. The Alcohol Use Disorders Identification Test–Consumption (AUDIT-C) [15] was used to assess risky alcohol drinking. The AUDIT-C is a well-validated instrument used to assess risky drinking in several ‘high-stress’ occupations, such as physicians, military personnel, firefighters, veterinary surgeons, and emergency department staff [16–20]. AUDIT-C scores were dichotomized into ‘non-risky drinking’ and ‘risky drinking’ categories with cutoff scores adjusted by gender (women ≥ 3; men ≥ 4) following established guidelines [21].

*Contemplating leaving the legal profession due to burnout*. The following item was used to assess whether participants contemplated leaving the profession due to mental health, burnout, or stress: “Are you considering, or have you left the legal profession due to mental health problems, burnout, or stress?” Participants responded “yes”, “no”, or “unsure”. “Unsure” responses were excluded from analyses.

### Data analysis

Demographic and mental health severity scores on the PHQ-9 and GAD-7 were compared between men and women using chi-square analyses. Logistic regression analyses were performed to identify associations between work-related predictor variables (Effort-Reward Imbalance Ratio, Work-Family Conflict, Work Overcommitment, Possibility of Promotion, Alcohol Permissiveness) and the outcome variables (stress, risky drinking, and contemplating leaving the profession) while controlling for covariates (COVID-19 impact, age, stress). Except for the COVID impact variable, all predictor variables were grouped into low, intermediate, and high tertiles.

Predictors were entered one at a time in a stepwise fashion, and their impact on the overall fit of the model was assessed. Those that significantly contributed to the model were entered into a final model along with the covariates of age and COVID-19 impact (e.g., single item added at the end of assessments that asked whether perceived problems increased, decreased, or stayed the same since COVID-19). COVID impact and age were entered as covariates in all models, and for models examining risky drinking and contemplating leaving due to burnout, a single item from the PSS was entered as a covariate to control for the influence of general stress (“In the last month, how often have you felt nervous and stressed?”). P-values for multiple comparisons were corrected using Holm-Bonferroni adjustments.

## Results

Of the 80,000 members of the CLA and D.C. Bar that were randomly selected and received a study invite, 5,292 consented, and 3,343 evaluable surveys were completed for a response rate of 6%. Of the evaluable surveys, 480 indicated they did not currently work in the legal profession and were removed from the final data analysis. The final sample consisted of 2,863 participants who indicated current employment in the legal profession.

### Descriptive results

#### Demographic variables

Women comprised approximately 51% (N = 1,473) of the sample. Table 1 shows the demographics of the participant sample. The sample of women tended to be younger. In addition, a significantly greater proportion of women (vs. men) were Asian or Pacific Islander (7.4% vs. 4.8%) or Black/African American (7.4% vs. 3.6%), while a significantly greater proportion of men were white (83.9% vs. 77.2%). Women were significantly less likely to be married (75.3% vs. 58.3%), were more likely to be divorced (10.5% vs. 7.9%) or never married (21.4% vs. 9.3%), and were less likely to have children compared to men (51.4% vs. 69.3%).

**Table 1:** Sample demographics.

	Women		Men	
	N	%	N	%
**Age**				
≤30	173	11.7%*	84	6.0%
31–40	411	27.9%*	326	23.5%
41–50	371	25.2%*	266	19.2%
51–60	315	21.4%	316	22.8%
61–70	175	11.9%	283	20.4%*
71 or older	28	1.9%	114	8.2%*
*Total N*	1473		1389	
**Race**				
Asian or Pacific Islander	109	7.4%*	67	4.8%
Black/African American	108	7.4%*	50	3.6%
Caucasian/White	1133	77.2%	1159	83.9%*
Latino/Hispanic	48	3.3%	58	4.2%
Native American	3	0.2%	3	0.2%
More than one race or Other	66	4.5%	45	3.3%
*Total N*	1467		1382	
**Relationship Status**				
Married	857	58.3%	1046	75.3%*
Widowed, Divorced, or Separated	154	10.5%*	110	7.9%
In a domestic partnership or civil union, or Single, but cohabitating with significant other	145	9.9%*	104	7.5%
Single, never married	314	21.4%*	129	9.3%
*Total N*	1470		1389	
**Children**				
No	712	48.6%*	426	30.7%
Yes	753	51.4%	960	69.3%*
*Total N*	1465		1386	

* Chi-Square Significant gender difference.

#### Work-related demographics

Work-related sample demographics are shown in Table 2. Approximately 67% of both women and men reported working over 40 hours in a typical workweek. Men tended to be in more senior legal positions than women and were also more likely to be in legal positions that involved litigation.

**Table 2:** Work-related demographics.

	Women		Men	
	N	%	N	%
**Hours worked in a typical week**				
Less than 10 hours to 30 hours	142	9.7%	151	11.0%
31 to 40 hours	342	23.4%	309	22.5%
41 to 50 hours	653	44.7%*	542	39.4%
51 to 71 or more hours	323	22.1%	373	27.1%*
*Total N*	1460		1375	
**Position in Legal Profession**				
Managing partner	214	15.6%	260	20.1%*
Senior partner	143	10.5%	218	16.8%*
Junior partner	79	5.8%	83	6.4%
Of counsel	105	7.7%	116	9.0%
Senior associate	205	15.0%	161	12.4%
Junior associate	188	13.7%*	122	9.4%
Clerk or paralegal	33	2.4%	18	1.4%
Other	401	29.3%*	316	24.4%
*Total N*	1368		1294	
**Position Involves Litigation**	843	57.7%	893	65.1%*

* Chi-Square Significant gender difference.

#### Mental health diagnoses and symptom severity

Approximately 80% of men and women indicated they were current drinkers, 7% were former drinkers, and 10% indicated being lifetime abstainers. A significantly greater proportion of men than women were current substance users (11.6% vs. 8.3%) or former substance users (15.2% vs. 10.3%). In comparison, women were significantly more likely to be lifetime abstainers from substances other than alcohol (81.5% vs. 73.2%).

Table 3 shows the proportions of attorneys within the severity ranges of the PHQ-9, GAD-7, PSS, and the AUDIT-C. A significantly greater proportion of women than men had PHQ-9 scores in the mild to moderately severe range. Similar results were reported with the GAD-7 and PSS, where a significantly greater proportion of women (vs. men) were in the mild (GAD-7 only), moderate, and severe ranges. A significantly greater proportion of women (vs. men) engaged in risky drinking (55.9% vs. 46.4%) and hazardous drinking (34.0% vs. 25.4%) according to the AUDIT-C.

**Table 3:** The severity and prevalence of depression, anxiety, stress, and drinking.

	Women		Men		Total	
	N	%	N	%	N	%
**PHQ-9 –Depression Symptoms**						
None/Minimal	642	43.6%	854	61.4%*	1496	52.3%
Mild	530	36.0%*	323	23.2%	853	29.8%
Moderate	202	13.7%*	148	10.6%	350	12.2%
Moderately Severe	77	5.2%*	44	3.2%	121	4.2%
Severe	22	1.5%	21	1.5%	43	1.5%
*Total N*	1473		1390		2863	
**GAD-7 –Anxiety Symptoms**						
None/Minimal	642	43.6%	840	60.4%*	1482	51.8%
Mild	500	33.9%*	349	25.1%	849	29.7%
Moderate	207	14.1%*	139	10.0%	346	12.1%
Severe	124	8.4%*	62	4.5%	186	6.5%
*Total N*	1467		1382		2863	
**PSS–Stress**						
Low	492	33.4%	713	51.3%*	1205	42.1%
Moderate	850	57.7%*	599	43.1%	1449	50.6%
Severe	131	8.9%*	78	5.6%	209	7.3%
*Total N*	1470		1390		2863	
**AUDIT-C–Risky Drinking**						
Yes	823	55.9%*	645	46.4%	1468	51.3%
No	650	44.1%	745	53.6%*	1395	48.7%
*Total N*	1473		1390		2863	
**AUDIT-C–Hazardous Drinking**						
Yes	500	34.0%*	353	25.4%	853	29.8%
No	973	66.0%	1037	74.6%*	2010	70.2%
*Total N*	1473		1390		2863	

* significant difference from referent (*p ≤ .05; **p ≤ .01; ***p ≤ .001); *OR* = odds ratio; *CI* = confidence interval.

#### Occupational stress, work-family conflict, and permissiveness toward alcohol in the workplace (Your Workplace)

Women had a significantly higher ERI score that reflected greater effort needed for reward (Mean = 1.04, SD = .42) compared to men who had a score that reflected less effort needed for reward (Mean = .96, SD = .43). Women also had a significantly higher overcommitment score compared to men (Mean = 15.19, SD = 3.72 vs. Mean = 14.12, SD = 3.77), a significantly higher Work-Family Conflict score (Mean = 6.72, SD = 2.76 vs. Mean = 6.23, SD = 2.61), and a significantly higher Your Workplace score (Mean = 18.56, SD = 5.54 vs. Mean = 17.82, SD = 5.75). Men, compared to women, had a higher likelihood of promotion score (Mean = 8.21, SD = 2.09 vs. Mean = 7.99, SD = 2.19). Comparing the proportion of women and men who scored above one on the ERI ratio (a reflection of effort-reward imbalance at work) revealed that roughly half of all women had an imbalance in the form of greater required effort (47.9%) compared to 38.7% of men. Additionally, one-quarter of all women in the sample indicated they had contemplated leaving the legal profession due to mental health or burnout, a proportion significantly greater than the proportion of men who contemplated leaving (17.4%).

### Logistic regression

#### Stress

Table 4 depicts the results of the logistic regression analysis examining predictors of moderate to high levels of perceived stress. Primary significant predictors of stress in men included COVID effect on stress, age, work-family conflict, effort-reward imbalance, work overcommitment, and promotion. Men with high or intermediate (vs. low) work-family conflict were 2.43 (95% CI = 1.56–3.77) and 1.65 (95% CI = 1.19–2.27) times more likely to report moderate to high stress. Compared to men with low effort-reward imbalance, those with high effort-reward imbalance were 2.24 (95% CI = 1.47–3.41) times more likely to have moderate or high stress. Men who reported high or intermediate (vs. low) work overcommitment were 4.63 (95% CI = 3.02–7.14) and 1.93 (95% CI = 1.39–2.68) times more likely to have moderate or high stress. Compared to men 61 or older, those who were 41 or below and 41 to 60 were 3.91 (95% CI = 2.69–5.67) and 2.30 (95% CI = 1.64–3.21) times more likely, respectively, to have moderate or high stress. Compared to men who reported a decrease or no effect of COVID on stress, those who reported an increase were 2.79 times more likely to contemplate leaving (95% CI = 2.14–3.64). The likelihood of promotion had an inverse relationship with stress. Compared to men with low scores on the promotion subscale, those with high or intermediate scores were 2.36 times less likely (95% CI = 1.50–3.53) and 1.64 times less likely (95% CI = 1.05–2.02), respectively, to have moderate or high stress (ORs and CIs divided by 1 for ease of interpretation).

**Table 4:** Work-related predictors of stress.

	Women (N = 1,471)			Men (N = 1,387)		
	N	OR	95% CI	N	OR	95% CI
**COVID–stress**		p < .0001			p < .0001	
No Change/Decrease	510(34.7%)			683(49.2%)		
Increase	961(65.3%)	4.097***	(3.14–5.35)	704(50.8%)	2.789***	(2.14–3.64)
**Age**		p < .0001			p < .0001	
Less than 40	584(39.7%)	2.264***	(1.51–3.40)	410(29.6%)	3.905***	(2.69–5.67)
41 to 60	684(46.5%)	1.194	(.81–1.75)	581(41.9%)	2.296***	(1.64–3.21)
61 and older	203(13.8%)			396(28.6%)		
**Alc. permissiveness at workplace**		p = .301			p = .283	
Low	462(31.4%)			513(37.0%)		
Intermediate	462(31.4%)	1.279	(.93–1.77)	442(31.9%)	.770	(.56–1.06)
High	547(37.2%)	1.207	(.87–1.67)	432(31.1%)	.871	(.62–1.22)
**Work-Family Conflict**		p = .203			p < .0001	
Low	589(40.0%)			632(45.6%)		
Intermediate	458(31.1%)	1.278	(.93–1.76)	459(33.1%)	1.647**	(1.19–2.27)
High	424(28.8%)	1.383	(.91–2.10)	296(21.3%)	2.425***	(1.56–3.77)
**Effort-Reward Imbalance**		p < .0001			p = .001	
Low	395(26.9%)			515(37.1%)		
Intermediate	524(35.6%)	1.955***	(1.42–2.70)	455(32.8%)	1.357	(.97–1.89)
High	552(37.5%)	2.387***	(1.58–3.61)	417(30.1%)	2.241***	(1.47–3.41)
**Work Overcommitment**		p < .0001			p < .0001	
Low	351(23.9%)			476(34.3%)		
Intermediate	572(38.9%)	1.846***	(1.33–2.55)	535(38.6%)	1.930***	(1.39–2.68)
High	548(37.3%)	5.134***	(3.34–7.88)	376(27.1%)	4.639***	(3.02–7.14)
**Possibility of Promotion**		p < .0001			p < .0001	
Low	570(38.7%)			458(33.0%)		
Intermediate	569(38.7%)	.604**	(.44-.84)	588(42.4%)	.687*	(.50-.95)
High	332(22.6%)	.449***	(.31-.66)	341(24.6%)	.423***	(.28-.63)

* significant difference from referent (*p ≤ .05; **p ≤ .01; ***p ≤ .001); *OR* = odds ratio; *CI* = confidence interval.

Primary/significant predictors of moderate to high perceived stress in women included COVID effect on stress, age, effort-reward imbalance, work overcommitment, and promotion. For women, work overcommitment had the highest odds ratio regarding association with having moderate or severe stress. Compared to women with low effort-reward imbalance, those with intermediate and high effort-reward imbalance were 1.96 (95% CI = 1.41–2.70) and 2.39 (95% CI = 1.59–3.61) times more likely to have moderate or high stress. Women 41 and below were 2.26 (95% CI = 1.51–3.40) times more likely to have moderate or severe stress than women 61 and older. Compared to women who reported a decrease or no effect of COVID on stress, those who reported an increase in stress due to COVID were 4.10 times more likely to have moderate or severe stress (95% CI = 3.14–5.35). Compared to women who perceived a low possibility of promotion, women who perceived a high possibility of promotion were 2.23 times less likely (95% CI = 1.52–3.27) to have moderate or severe stress, and those with intermediate possibility of promotion were 1.66 times less likely (95% CI = 1.19–2.30) (ORs and CIs divided by 1 for ease of interpretation).

#### Risky drinking

Table 5 depicts the results of the logistic regression analysis examining predictors of whether someone endorsed AUDIT-C risky drinking (adjusted for gender). Primary predictors of risky drinking for both men and women included workplace permissiveness toward alcohol and COVID impact. Overcommitment was a predictor of risky drinking in men but not women. For men, the likelihood of risky drinking was 1.71 times higher (95% CI = 1.26–2.33) for those scoring high on alcohol permissiveness at work (vs. low). Men who reported intermediate (vs. low) work overcommitment were 1.43 times more likely (95% CI = 1.06–1.92) to engage in risky drinking. Compared to men who reported a decrease or no effect of COVID on drinking, those who reported an increase in drinking due to COVID were 3.73 times more likely to engage in risky drinking (95% CI = 2.81–4.96).

**Table 5:** Work-related predictors of risky drinking.

	Women (N = 1,312)			Men (N = 1,237)		
	N (%)	OR	95% CI	N	OR	95% CI
**COVID–drinking**		p < .0001			p < .0001	
No Change/Decrease	858(65.4%)			876(70.8%)		
Increase	454(34.6%)	6.993***	(5.13–9.53)	361(29.2%)	3.734***	(2.81–4.96)
**Age**		p = .053			p = .051	
Less than 40	548(41.8%)	.632*	(.43-.94)	380(30.7%)	1.211	(.86–1.71)
41 to 60	591(45.0%)	.642*	(.44-.94)	515(41.6%)	.846	(.621–1.15)
61 and older	173(13.2%)			342(27.6%)		
**Alc. permissiveness at workplace**		p = .038			p = .002	
Low	387(29.5%)			436(35.2%)		
Intermediate	416(31.7%)	.957	(.70–1.30)	393(31.8%)	1.369*	(1.03–1.83)
High	509(38.8%)	1.373*	(1.01–1.87)	408(33.0%)	1.714**	(1.26–2.33)
**Stress**					p = .402	
Low				309(25.0%)		
Intermediate				490(39.6%)	.802	(.58–1.11)
High				438(35.4%)	.880	(.60–1.28)
**Work-Family Conflict**						
Low						
Intermediate						
High						
**Effort-Reward Imbalance**						
Low						
Intermediate						
High						
**Work Overcommitment**		p = .533			p = .048	
Low	308(23.5%)			413(33.4%)		
Intermediate	507(38.6%)	.956	(.70–1.31)	483(39.0%)	1.428*	(1.06–1.92)
High	497(37.9%)	1.120*	(.81–1.55)	341(27.6%)	1.142	(.80–1.63)
**Possibility of Promotion**						
Low						
Intermediate						
High						

*significant difference from referent (*p ≤ .05; **p ≤ .01; ***p ≤ .001); *OR* = odds ratio; *CI* = confidence interval.

For women, the only predictors significantly associated with risky drinking were alcohol permissiveness at work and COVID effect on drinking. Women with high (vs. low) workplace permissiveness toward alcohol were 1.37 times more likely to engage in risky drinking (95% CI = 1.01–1.87). Compared to women who reported a decrease or no effect of COVID on drinking, those who reported an increase in drinking were 6.99 times more likely to engage in risky drinking (95% CI = 5.13–9.53).

#### Leaving the legal profession

Table 6 depicts the results of the logistic regression analysis examining predictors of whether someone indicated yes or no to the question, “Are you considering, or have you left the legal profession due to mental health problems, burnout, or stress?”. For men, the likelihood of contemplating leaving the job was 4.46 times higher (95% CI = 2.27–8.74) for those with high (vs. low) self-reported stress and was 2.36 times higher (95% CI = 1.23–4.53) for those with intermediate (vs. low) stress. Additionally, men with high or intermediate (vs. low) work-family conflict were 2.47 (95% CI = 1.47–4.17) and 1.78 (95% CI = 1.12–2.82) times more likely, respectively, to report contemplating leaving. Men who reported high (vs. low) work overcommitment were 2.38 times more likely (95% CI = 1.36–4.14) to contemplate leaving. Men 41 or below were 2.26 times more likely to contemplate leaving (95% CI = 1.37–3.72) compared to men 61 and older. Compared to men who reported a decrease or no effect of COVID on anxiety, those who reported an increase in anxiety due to COVID were 1.40 times more likely to contemplate leaving (95% CI = 1.00–1.96). Perceived likelihood of promotion had an inverse relationship to contemplating leaving on men. Compared to men with low promotion scores, those with high or intermediate scores were 2.46 times less likely (95% CI = 1.47–4.10) and 1.64 times less likely (95% CI = 1.12–2.40) to contemplate leaving the profession (ORs and CIs divided by 1 for ease of interpretation).

**Table 6:** Work-related predictors of leaving or contemplating leaving the legal profession.

	Women (N = 1,346)			Men (N = 1,277)		
	N (%)	OR	95% CI	N	OR	95% CI
**COVID–anxiety**		p = .004			p = .049	
No Change/Decrease	456(33.9%)					
Increase	890(66.1%)	.639**	(.47-.87)	593 (46.4%)	.715*	(.51-.999)
**Age**		p < .0001			p = .004	
Less than 40	533(39.6%)	3.496***	(1.99–6.13)	375(29.4%)	2.264**	(1.38–3.72)
41 to 60	626(46.5%)	3.054***	(1.76–5.32)	532(41.7%)	1.623	(1.00–2.64)
61 and older	187(13.9%)			370(29.0%)		
**Stress**		p = .001			p < .0001	
Low	148(11.0%)			329(25.8%)		
Intermediate	512(38.0%)	1.028	(.58–1.83)	507(39.7%)	2.364**	(1.23–4.53)
High	686(51.0%)	1.824*	(1.02–3.25)	441(34.5%)	4.456***	(2.27–8.74)
**Alc. permissiveness at workplace**						
Low						
Intermediate						
High						
**Work-Family Conflict**		p < .0001			p = .003	
Low	558(41.5%)			590(46.2%)		
Intermediate	414(30.8%)	1.766**	(1.21–2.59)	421(33.0%)	1.779*	(1.12–2.81)
High	374(27.8%)	4.650***	(3.09–7.00)	266(20.8%)	2.471**	(1.47–4.17)
**Effort-Reward Imbalance**					p = .453	
Low				477(37.4%)		
Intermediate				423(33.1%)	.758	(.47–1.23)
High				377(29.5%)	.913	(.54–1.56)
**Work Overcommitment**		p = .078			p = .001	
Low	322(23.9%)			437(34.2%)		
Intermediate	528(39.2%)	1.500	(.95–2.37)	504(39.5%)	1.218	(.74–2.02)
High	496(36.8%)	1.788*	(1.08–2.96)	336(26.3%)	2.376**	(1.36–4.14)
**Possibility of Promotion**					p = .002	
Low				420(32.9%)		
Intermediate				544(42.6%)	.610*	(.42-.89)
High				313(24.5%)	.407**	(.24-.68)

*significant difference from referent (*p ≤ .05; **p ≤ .01; ***p ≤ .001); *OR* = odds ratio; *CI* = confidence interval.

For women, work-family conflict had the highest odds ratio with regard to association with contemplating leaving the legal profession due to mental health, stress, or burnout. More specifically, compared to women with low work-family conflict, those with high work-family conflict were 4.60 times more likely to contemplate leaving (95% CI = 3.09–7.01). Women 40 or below and 41 to 60 were 3.50 (95% CI = 1.99–6.13) and 3.05 (95% CI = 1.76–5.32) times more likely to contemplate leaving than women 61 and older. Additionally, women with high stress (vs. low) were 1.82 times more likely to contemplate leaving (95% CI = 1.02–3.25). Compared to women who reported a decrease or no effect of COVID on anxiety, those who reported an increase in anxiety due to COVID were 1.56 times more likely to contemplate leaving (95% CI = 1.15–2.12). In contrast to men, promotion was not associated with leaving the profession in women.

## Discussion

The present study provides insight into factors associated with the experiences of stress, risky drinking, and attrition in the legal profession. An overarching finding was that men and women differ with respect to both the prevalence of these problems and the degree to which workplace factors may contribute to them.

In the present study, younger attorneys were 2–4 times more likely than their older colleagues to report moderate or high stress. This finding is consistent with what has been observed in other high-stress professions, such as medicine, where younger age is a significant factor associated with physician burnout [22]. For the legal profession, this is noteworthy and should inform a variety of domains, from the development of mitigation strategies to the identification, allocation, and targeted deployment of supports, resources, tools, and training. The fact that younger attorneys experience significantly higher levels of stress also suggests an increased role for law schools in better equipping their students for the experiences that lie ahead. Some progress has been made in this realm, and a recent survey of law school efforts to improve mental health suggests that a handful of schools have emerged as trailblazers in this arena, but others still have considerable work to do [23].

Depending upon the specific employment context, the origins of a lawyer’s workload may vary in nature, from high or possibly unrealistic productivity requirements set by an employer to the practical demands, such as generating enough revenue to simply stay afloat, often faced by solo practitioners. Heavy workloads and overcommitment were reflected in the sample of the present study. For example, 67% of the sample reported working over 40 hours per week, and nearly a quarter indicated working over 51 hours per week on average. Furthermore, overcommitment scores, as assessed by the ERI Questionnaire, were similar to scores reported in other high-stress occupations (e.g., doctors, nursing, and law enforcement) [24–26]. Findings from other studies indicate that overcommitment is associated with a higher prevalence of psychiatric distress [27] and that this association is higher among women than men [28]. Our findings align with these reports and demonstrate that while high (vs. low) work overcommitment was strongly associated with stress among both sexes, this relationship was strongest in women. Hard work and professional rigor have long been associated with the life of a practicing lawyer. However, there is a point where workloads become untenable, threatening to diminish the health and well-being of those tasked with supporting them. Excessive workloads also have the potential to undermine the quality and reliability of the work product delivered in their service since chronic stress has been consistently associated with lower cognitive function [29].

Approximately 30% of our sample screened positive for high-risk hazardous drinking according to the AUDIT-C (≥ 4 for women and ≥ 5 for men), which is interpreted to be within the range of alcohol abuse or possible alcohol dependence [15,30]. Despite the high prevalence of hazardous drinking as assessed by the AUDIT-C, we were struck by the low prevalence of attorneys who self-reported ever having received an Alcohol Use Disorder diagnosis (2% of the sample). This disparity suggests an extreme level of underdiagnosis and treatment for a widespread problem, possibly owing to pervasive denial, stigma, and a professional culture that normalizes heavy drinking.

An additional noteworthy finding regarding alcohol use is that a significantly greater proportion of women compared to men engaged in risky drinking (55.9% vs. 46.4%) and high-risk/hazardous drinking (34.0% vs. 25.4%). This finding is at odds with several other studies outside the legal profession indicating that men typically exceed women in terms of problematic alcohol use as defined by the AUDIT-C [31,32]. However, it supports previous reports within the legal profession, indicating heightened problematic drinking in women compared to men [1]. This finding, along with the fact that women also had elevated levels of anxiety, depression, and stress, highlights a very real mental health disparity that exists within the legal profession. Identifying why women in the legal profession are suffering disproportionately requires ongoing and sustained attention.

Over 80% of the attorneys considered themselves a current drinker. In contrast, an estimated 55% of the U.S. population drank in the past month, and an estimated 70% drank in the last year [33]. Over half of the lawyers screened positive for risky drinking on the AUDIT-C, and 30% screened for high-risk hazardous drinking. Findings from the present study indicated that workplace permissiveness towards alcohol use was a primary predictor of risky drinking among men and women. This finding supports the perception of an alcohol-based social culture that has long typified the legal profession [34]. In the absence of historical or longitudinal data on the association between risky drinking and workplace permissiveness towards alcohol use in the legal profession, we cannot determine whether this association has been weakened in recent years because of ongoing calls for the deemphasis of alcohol within the profession. However, we can conclude that this association continues to exist and thereby merits additional and sustained efforts to emancipate the practice of law from a pervasive expectation of alcohol use.

Compared to men who reported a decrease or no effect of COVID on drinking, those who reported an increase in drinking due to COVID were almost four times more likely to engage in risky drinking. Women who reported an increase in drinking due to COVID were seven times more likely to drink riskily. These inauspicious findings may signal the early manifestation of what will ultimately prove to be a long-term problem for some lawyers. Although we did not probe the specific reasons why respondents were drinking more in response to COVID, it is reasonable to conclude that many were drinking more because of heightened anxiety and stress associated with the pandemic, and research has shown that drinking to cope with negative affect and anxiety can greatly increase the risk of persistent alcohol dependence [35]. This finding highlights the importance of helping lawyers develop healthy coping skills to reduce the likelihood of resorting to alcohol in times of high stress.

Considering the higher rates of mental health distress experienced by female attorneys, an expected but troubling result is that more women than men (24.2% vs. 17.4%) contemplated leaving the legal profession due to mental health problems, burnout, or stress. This is an undesirable outcome for a profession long bedeviled by its inability to retain female attorneys [5,36–39] and raises the question of whether improving workplace factors that influence poor mental health might be an important missing ingredient in those efforts.

Predictors of leaving the profession due to mental health or burnout differed between women and men. The workplace-related factor most predictive of contemplating leaving the profession for women was work-family conflict. Women with a high work-family conflict score were roughly 4.5 times more likely to leave or consider leaving the profession due to mental health, burnout, and stress. Work-family conflict was also a significant factor for men, albeit less so. This aligns with findings from a large ABA-sponsored survey in which more than half of the women indicated that caretaking commitments or work-family conflict were a primary reason for leaving their firm [36]. Notably, more men than women report being married with children, perhaps suggesting that anticipation of work-family conflict may also influence the decisions of female attorneys about whether, or when, to marry or otherwise establish a family unit in the first place. The possibility that work-family conflict is influencing decisions about marriage is also relevant to our findings that women are experiencing worse mental health than men since married adults, and to a lesser extent, those in non-marital committed partnerships, have shown better psychological well-being than their single counterparts in samples from nearly two dozen countries [40]. Overall, our findings related to work-family conflict align with research in other industries and professions such as banking, pharmaceuticals, medicine, science, and engineering, in which high-work family conflict was either directly or indirectly associated with job dissatisfaction and turnover intentions [41,42].

Work overcommitment was also a significant predictor of leaving the profession due to mental health, burnout, or stress among men, and it approached significance in women. In fact, men with high work overcommitment were more than twice as likely to contemplate leaving the profession due to mental health, and women with high overcommitment were 1.78 times more likely to leave. This is an unsurprising but unfortunate outcome that raises a question of how many otherwise talented lawyers and gifted legal minds have found themselves driven from the profession for reasons wholly unrelated to their skill, intellect, or passion for the law.

Finally, the perceived likelihood of promotion was associated with a lower likelihood of leaving or contemplating leaving the profession due to mental health, burnout, or stress for men. However, the same did not hold true for women. Specifically, men with high or intermediate scores on the perceived possibility of promotion subscale were approximately 2.5 times less likely to leave the profession due to mental health, but no association between these items was present for women. Therefore, it would seem that whatever benefit the perceived possibility of promotion is affording men as it relates to mental health, burnout, or stress is not transferring equally to women. One could speculate that women frequently anticipate less opportunity or chance for promotion, thereby rendering that possibility less relevant to their calculation about whether to leave the profession due to mental health. Reports from the field lend strong support to this, with one survey indicating that 53% of women indicated being denied or overlooked for advancement or promotion compared to only 7% of men [36]. It could also be surmised that, on balance, female attorneys do not view the possibility of promotion as being meaningful or important enough to offset their concerns about mental health, stress, and burnout. It is likely that both factors, along with others, could account for this finding.

### Limitations

We did not examine help-seeking motives and behaviors and are therefore unable to opine whether progress has been made in encouraging lawyers to seek help for their struggles when needed, though much effort has been directed toward that goal, and anecdotal evidence would indicate at least some improvement. Additionally, as mentioned, the survey occurred during a national crisis, the COVID-19 pandemic. While efforts were made to assess the extent COVID may have influenced the results of the present study, it is expected that the impact occurred in ways unaccounted for in the design of the study and in the accuracy of reporting from the participants. It is quite possible that despite stating that mental health symptoms did not change since the beginning of the pandemic, such changes may have gone unnoticed in some respondents. While this could be a limitation of all survey-based studies, it could be argued that accurate assessment of whether a major event influenced a single symptom would require an inordinate level of self-awareness. An additional limitation relates to the wording of the COVID items. The items asked whether participants believed their problems increased, decreased, or stayed the same since COVID. It is reasonable to assume that COVID-19 was a major factor; however, other life events or situations that occurred during this time but were unrelated to the pandemic may have also contributed to their response.

## Conclusion

Our findings raise meaningful concerns about the stress levels of both men and women and the possible impact of that stress on the delivery of effective legal services. Ultimately, when a client hires an attorney or law firm, they expect that the individuals representing them are not experiencing cognitive impairment or diminished executive function due to job burnout. In a profession where work overcommitment appears both rampant and significantly predictive of high stress, it would be reasonable to question how consistently those client expectations are being met and whether more safeguards are warranted to facilitate less overcommitment across a variety of legal work environments. Professional training and interventions that have proven effective in addressing burnout among physicians could be considered for the legal profession, such as cognitive behavioral therapy, monthly meetings focused on work-life and personal challenges, offloading non-essential tasks to staff, standardizing and synchronizing workflows, stress reduction activities, and adherence to limitations in work hours [43]. Additionally, physicians who engage in problematic drinking or experience other substance use disorder problems often receive support through Physician Health Programs and, when necessary, are required to achieve abstinence and stay under monitoring for several years. Lawyer Assistance Programs play a similar role in the legal profession, providing both support for and, in some instances, monitoring of attorneys with substance use disorders. Greater familiarity with these programs and the services they offer to the legal profession is warranted.

Furthermore, a career in law should not be antagonistic to the full expression of a lawyer’s humanity, including their ability to undertake and navigate familial obligations should they so desire. Strategies and interventions aimed at alleviating work-family conflict would be wise pursuits for legal employers hoping to reduce unwanted turnover and increase the likelihood that their attorneys will be able to thrive across all dimensions of their lives. Findings from the present study also revealed an inverse relationship between the perceived likelihood of promotion and perceived stress, suggesting that possibility of promotion is likely a protective factor against perceived stress. Unfortunately, the business models of many legal employers, as well as the pyramidical or hierarchical structures of many employment settings generally, would seem to necessarily limit the availability of this protective factor by predetermining the number of possible promotions, often through an “up or out” system. As such, employers may be able to reduce perceived stress by pursuing creative solutions to widen the range of career tracks and opportunities for growth currently available to their lawyers.

Finally, it is clear from our data that workplace attitudes and permissiveness towards alcohol significantly influence the likelihood of problematic drinking among attorneys. Changing workplace attitudes towards alcohol is an ostensibly straightforward solution for reducing the incidence of problem drinking that will nonetheless continue to be challenging. Given the cultural embrace and seeming omnipresence of alcohol within law firm gatherings and other professional events, the goal of changing attitudes is likely to be best accomplished through sustained, incremental efforts. An essential component of those efforts should be education, as educational interventions and the provision of structured advice about drinking behaviors have been widely shown to reduce problematic drinking in a variety of populations [44–46].

In conclusion, our research identifies key areas upon which stakeholders in the legal profession should focus their efforts to improve lawyer mental health and well-being. Overall, findings from the present study suggest that levels of mental health problems and problematic drinking continue to be quite high among currently employed attorneys. Women experience more mental health distress, greater levels of overcommitment and work-family conflict, and lower prospects of promotion than men in the legal profession and are more likely to leave as a result. Addressing the structural, cultural, and organizational infrastructures responsible for this mental health gender disparity will be an important step towards achieving the profession’s longstanding goals around the retention of female attorneys.
